# Association Between Self-Reported Sitting Time and the Physical Function Domain of the Kihon Checklist Among Community-Dwelling Older Adults

**DOI:** 10.7759/cureus.74705

**Published:** 2024-11-28

**Authors:** Taishiro Kamasaki, Mizuki Hachiya, Hiroyuki Okawa, Kazuhiko Fujiwara, Kodai Hosaka, Takuya Suenaga, Yo Kichize, Yasuhiro Mizokami, Minoru Kamata, Hiroshi Otao

**Affiliations:** 1 Department of Rehabilitation Sciences, Nishikyushu University, Saga, JPN; 2 Department of Rehabilitation, Medical Corporation Kabutoyamakai Kurume Rehabilitation Hospital, Fukuoka, JPN; 3 Department of Rehabilitation Medicine, Keitendo Koga Hospital, Saga, JPN; 4 Department of Rehabilitation, St. Mary’s Hospital, Fukuoka, JPN; 5 Department of Research and Development, Miz Co. Ltd, Saga, JPN; 6 Department of Community Healthcare, Suwa Central Hospital, Nagano, JPN

**Keywords:** community-dwelling older adults, physical activity, physical function, sitting time, stair climbing

## Abstract

Objective

Interventions that reduce sitting time are easier to implement than those that aim to increase physical activity in compliance with the guidelines. There is no consensus on the association between sitting time as assessed by the International Physical Activity Questionnaire (IPAQ) and physical function. We investigated the association between self-reported sitting time and physical function according to the Kihon Checklist (KCL) among community-dwelling older adults.

Methods

This cross-sectional study included 87 community-dwelling middle-aged and older adults who had participated in a long-term preventive care and health promotion project. The project was conducted three times between March 2023 and March 2024. Sitting time was self-reported using the IPAQ. Physical function was assessed using the KCL. A generalized linear model was used to analyze the association between sitting time and physical function. In the adjusted model, handgrip strength, walking speed, five times sit to stand test, skeletal muscle mass index, living alone, pain, sex, and age were used as covariates to adjust for confounders.

Results

Eight subjects met the exclusion criteria, leaving a final cohort of 79 community-dwelling older adults (mean age: 75 ± 6 years, 73% female). Our analysis revealed that sitting time was associated with physical function, even after adjusting for covariates (standardized β coefficient = 0.22, p = 0.023).

Conclusions

Our findings emphasize the usefulness of assessing sitting time to maintain physical function in community-dwelling older adults. Furthermore, it may be important to reduce sitting time to maintain or improve physical function.

## Introduction

The increasing aging population worldwide has led to a growing demand for research on preventive care and health promotion. Resistance training improves physical function in older adults [1], and dietary interventions have been reported to effectively prevent hypertension [2] and maintain or improve physical function and muscle strength [3]. Although these interventions have beneficial effects, they may be difficult to sustain due to time and financial constraints.

Physical activity (PA) has recently attracted attention from the perspectives of preventive care and health promotion. PA can be increased through activities of daily living (ADL) and is considered less time-consuming and expensive than resistance training and dietary interventions. PA has a health-protective effect. For example, a lack of PA is a risk factor for chronic diseases, such as heart disease, diabetes mellitus, and cancer [4,5], and PA levels affect the risk of mortality [6]. Guidelines for achieving beneficial health outcomes of PA recommend at least 150 minutes of moderate-intensity aerobic exercise or 75 minutes of high-intensity aerobic exercise per week and at least two days of muscle-strengthening activity [7]. However, one must also consider that older adults may have difficulty achieving the PA recommended in these guidelines [8]. Thus, more emphasis has been placed on sitting time in this specific population. Sedentary behavior is ranked as the lowest level of PA [9], and interventions that reduce sitting time are easier to implement than those that aim to increase PA in compliance with the guidelines.

There has been a recent increase in reports on sitting time among older adults in recent years. Sitting time in older adults is associated with frailty, which has important implications for preventive care and health promotion [10], and with quality of life [11]. Through these studies, the association between sitting time and health status and clinical outcomes in older adults is becoming clearer. On the other hand, it was reported that there was no association between sitting time and physical function among community-dwelling older adults [12]. Another study found that although there was an association between sitting time and physical function, its strength was weakened when covariates were included [13]. However, because sitting time is associated with health status and clinical outcomes [10,11,14], we believe there may also be an association between sitting time and physical function.

Thus, the objective of this study was to investigate the association between self-reported sitting time and physical function among community-dwelling older adults. It will also identify which of the physical functions and performances related to ADL correlate with sitting time. We believe that our findings contribute to the maintenance and improvement of the physical functions of community-dwelling older adults, which, in turn, contributes to health promotion and preventive care based on sitting time. Overall, we aimed to demonstrate the importance of assessing and intervening in the level of PA in community-dwelling older adults, particularly in terms of sitting time.

## Materials and methods

Participants

The participants of this cross-sectional study were community-dwelling older adults who participated in physical fitness tests to prevent long-term care and improve their health. The physical fitness tests were conducted three times between March 2023 and March 2024. The participants were recruited by posting on the website, posters, and staff members speaking to community residents. Participants were excluded if they were aged ≤64 years, had pain that made measurement difficult, required walking assistance, had cognitive decline, or had missing data. All participants in the physical fitness test were fully informed of the study’s content and purpose and gave their consent and agreed to cooperate. Additionally, participants were allowed to participate in the physical fitness test even if they did not consent to the study, and it was explained that withholding consent would not be viewed in a negative light. The study was approved by the Ethical Review Committee of Nishikyushu University (approval number: 24PBV09, approval date: May 31, 2024).

Measured items

Basic patient information (sex, age, family structure, and pain during PA) was recorded. Height, weight, and skeletal muscle mass index (SMI) were measured. Physical function was assessed using the Kihon Checklist (KCL) and self-reported sitting time. The tests performed included handgrip strength, walking speed, five times sit to stand test (FTSST), open-eyed one-leg standing (OLS) test, and timed up and go (TUG) test. The cognitive state was assessed using the mini-mental state examination (MMSE). All measurements were performed by licensed physical and occupational therapists and by physical and occupational therapy students who received lectures on measurement knowledge and skills.

Physical function

Physical function was assessed by KCL. This questionnaire was developed by the Japanese Ministry of Health, Labor, and Welfare and is a multidimensional method for evaluating ADL [15]. Due to its usefulness, it has also been used in other countries besides Japan [16]. The KCL consists of 25 questions in seven domains: instrumental ADL, physical function, nutritional status, oral function, social isolation, cognitive function, and depressed mood (Appendices). The higher the total score, the higher the likelihood of functional decline. We assessed physical function by calculating the total score from the responses to subitems 6-10 (physical function domain) of the KCL (Do you normally climb stairs without using a handrail or wall for support? Do you normally stand up from a chair without any aids? Do you normally walk continuously for 15 minutes? Have you experienced a fall in the past year? Do you have a fear of falling while walking?) according to a previous study.

Sitting time

Sitting time was self-reported using the International Physical Activity Questionnaire (IPAQ). The respondents were asked how much time they spent sitting or lying down each day. To avoid misunderstandings, we explained that they should recall the past week and answer with the average time, excluding the time spent sleeping. IPAQ is the global standard for evaluating sitting time [17].

Other measurement items

Handgrip strength was measured using a Smedley grip strength meter (T.K.K. 3364, Takei Scientific Instruments Co., Ltd., Niigata, Japan). Grip strength was measured while the subject was in a standing position with the elbow joint of the measured limb extended. Measurements were taken twice, alternating left and right, and the maximum value was used for analysis. Grip strength values obtained using a Smedley grip strength meter are highly reliable [18].

Gait speed was measured using a digital stopwatch. Participants were instructed to walk at their usual speed, and the time required to walk the middle 5 m of a walking path of 11 m was recorded. Gait speed measurement using a digital stopwatch is highly reliable [19].

The FTSST was measured using a chair with a seat height of 40 cm and a digital stopwatch. The starting position was sitting with the upper limbs crossed in front of the chest. Participants were instructed to stand up five times as quickly as possible, and the time was measured. The FTSST is a reliable evaluation method [20].

The OLS was measured using a digital stopwatch. Participants were instructed to hold a standing position on one leg. The termination criteria were as follows: both feet touching the ground, need for assistance, raised leg touching the opposite leg, and movement of the supporting leg. The upper limit was set at 120 seconds. The test was performed once on each leg. The maximum value was used for analysis.

The TUG was measured using a digital stopwatch. Participants were instructed to stand up from a chair with a seat height of 40 cm, walk around a marker that was 3 m in front of them, and then sit back down in the chair. The test was performed twice. The shortest time was used for analysis.

The cognitive state was assessed using the MMSE questionnaire. The MMSE is the global standard for assessing cognitive function in older adults. The questionnaire comprises 11 items, and the higher the score on a 30-point scale, the higher the level of cognitive function.

Statistical analysis

The association between sitting time and physical function was statistically analyzed using a generalized linear model with the total points scored for the KCL physical function domain as the dependent variable and sitting time as the independent variable. In the adjusted model (Model 2), covariates (handgrip strength, gait speed, FTSST, SMI, living alone, pain, sex, and age) were used to adjust for confounders. An analysis of variance was used to confirm the significance of the generalized linear model. The goodness of fit, autocorrelation in residuals, and multicollinearity of the regression equations were checked using R2, the Durbin-Watson ratio, and the variance inflation factor (VIF), respectively. Furthermore, the correlation ratio between the sitting time and subitems comprising the physical function domain of the KCL was examined to confirm trends in the correlation between sitting time and physical performance in more detail. Statistical significance was set at 5% (p < 0.05). SPSS Statistics version 28.0 (IBM Corp. Released 2021. IBM SPSS Statistics for Windows, Version 28.0. Armonk, NY: IBM Corp.) was used for statistical analyses. Because the sample size could not be calculated a priori in this study, power was calculated posteriori using G*Power v3.1.9.7 (Heinrich-Heine-Universität Düsseldorf, Düsseldorf, Germany) in the generalized linear model.

## Results

Characteristics of the analyzed participants

**Figure 1:**
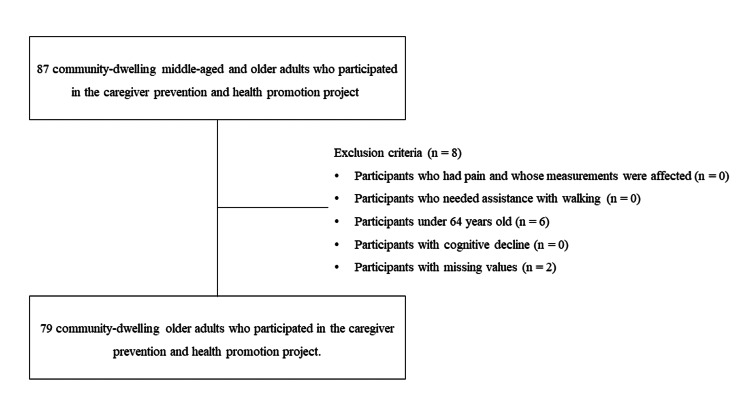
Analysis of the participant selection flowchart

Table 1 presents the characteristics of the 87 community-dwelling older adults who participated in the physical fitness tests. However, only 79 participants (mean age: 75 ± 6 years) were included in the final analysis because eight were excluded according to the exclusion criteria (Figure 1).

**Table 1:** Characteristics of the analyzed participants Mean ± SD, median (1st-3rd quartile), number (%) ^a^ t-test, ^b^ Mann-Whitney U test, ^c^ chi-square test, ^d^ Fisher's exact probability test, ^*^ Cohen's d, ^†^r, ^‡^ φ coefficient, ^§^ Cramer's V BMI: body mass index, SMI: skeletal muscle mass index, KCL: Kihon Checklist, FTSST: five times sit to stand test, OLS: open-eyed one-leg stand test, TUG: timed up and go test, MMSE: mini-mental state examination, ES: effect size, 95% CI: 95% confidence interval

		Overall (n = 79)	Male (n = 21)	Female (n = 58)	p-value	ES	95% CI (lower)	95% CI (upper)
Age	years	75 ± 6	77 ± 6	75 ± 6	0.115 ^a^	0.41 ^*^	-0.10	0.91
Height	cm	154.3 ± 6.6	162.0 ± 3.9	151.5 ± 5.0	< 0.001 ^a^	2.21 ^*^	1.60	2.81
Weight	kg	53.3 ± 9.0	61.0 ± 5.7	50.5 ± 8.4	< 0.001 ^a^	1.35 ^*^	0.80	1.89
BMI	kg/m^2^	22.3 ± 3.0	23.2 ± 2.0	22.0 ± 3.3	0.048 ^a^	0.42 ^*^	-0.09	0.92
SMI	kg/m^2^	6.4 ± 1.5	7.7 ± 2.2	6.0 ± 0.6	< 0.001 ^a^	1.41 ^*^	0.86	1.95
Pain (yes)	n (%)	36 (46%)	8 (38%)	28 (48%)	0.422 ^c^	0.09 ^‡^	-0.13	0.30
Living alone (yes)	n (%)	16 (20%)	2 (10%)	14 (24%)	0.212 ^d^	0.16 ^§^	0.14	0.32
Physical function	points	0 (0-1)	0 (0-1)	0 (0-1)	0.404 ^b^	0.09 ^†^	―	―
Sitting time	min	180 (120-270)	180 (120-270)	240 (120-280)	0.605 ^b^	0.06 ^†^	―	―
Handgrip strength	kg	26.6 ± 6.4	35.2 ± 3.8	23.4 ± 3.7	< 0.001 ^a^	3.16 ^*^	2.45	3.86
Gait speed	m/s	1.4 ± 0.3	1.3 ± 0.2	1.4 ± 0.3	0.221 ^a^	-0.31 ^*^	-0.82	0.19
FTSST	s	5.8 ± 2.0	6.0 ± 2.0	5.7 ± 2.0	0.302 ^a^	0.13 ^*^	-0.37	0.63
OLS	s	55 ± 43	42 ± 37	60 ± 45	0.081 ^a^	-0.42 ^*^	-0.93	0.10
TUG	s	5.9 ± 1.1	5.7 ± 1.1	6.0 ± 1.1	0.403 ^a^	-0.21 ^*^	-0.71	0.29
MMSE	points	29 ± 1	29 ± 1	29 ± 1	0.489 ^a^	0.18 ^*^	-0.32	0.68

The analyzed cohort included 21 males (mean age: 77 ± 6 years) and 58 females (mean age: 75 ± 6 years). All participants were asked to respond to a question about regular jobs that might have influenced their sitting time, but none of them had a regular job (Table 1).

Association between sitting time and KCL physical function

Table 2 presents the association between sitting time and KCL physical function. We used a generalized linear model with the KCL physical function domain score as the dependent variable and sitting time as the independent variable. This crude model revealed a significant association between the KCL physical function domain and sitting time (standardized β coefficient = 0.30, p = 0.008). Handgrip strength, walking speed, FTSST, SMI, living alone, pain, sex, and age were used as covariates in the adjusted model (Model 2). The results showed a significant association between the KCL physical function domain and sitting time (standardized β coefficient = 0.22, p = 0.023). The regression equation for Model 2 was significant (p < 0.001), with an R2 of 0.46 and a Durbin-Watson ratio of 2.47. Multicollinearity was confirmed by VIF, but no variable exceeded 5. The power of the adjusted model calculated posteriori was 1.00 (Table 2).

**Table 2:** Association between physical function of KCL and sitting time Generalized linear model Dependent variables: physical function of KCL Model 1: ANOVA = 0.008, R^2^ = 0.09, Durbin-Watson ratio = 2.18 Model 2: ANOVA < 0.001, R^2^ = 0.46, Durbin-Watson ratio = 2.47 SMI: skeletal muscle mass index, FTSST: five times sit to stand test, 95% CI: 95% confidence interval, VIF: variance inflation factor

		Non-standardization factor	95% CI (lower)	95% CI (upper)	Standardization factor	p-value	VIF
Crude model (model 1)	Sitting time	0.01	0	0.02	0.3	0.008	―
Adjustment model (model 2)	Sitting time	0.01	0	0.02	0.22	0.023	1.1
	Handgrip strength	-0.04	-0.09	0.01	-0.29	0.11	4.09
	Gait speed	-0.47	-1.21	0.27	-0.13	0.206	1.31
	FTSST	0.12	0.03	0.22	0.28	0.009	1.35
	SMI	0	-0.13	0.14	0.01	0.952	1.49
	Living alone (ref: no)	0.11	-0.3	0.52	0.05	0.579	1.13
	Pain (ref: no)	0.23	-0.1	0.56	0.13	0.172	1.12
	Sex (ref: male)	-0.19	-0.92	0.55	-0.1	0.612	4.42
	Age	0.05	0.02	0.08	0.31	0.004	1.37

Correlation between sitting time and KCL physical function domain subitems

Table 3 shows the correlation between the sitting time and KCL physical function domain subitems. The correlations with sitting time were as follows: η=0.44, p=0.045 for “Do you normally climb stairs without using a handrail or wall for support?”; η = 0.15, p = 0.599 for “Do you normally stand up from a chair without any aids?”; η = 0.17, p = 0.472 for “Do you normally walk continuously for 15 minutes?”; η = 0.18, p = 0.013 for “Have you experienced a fall in the past year?”; and η = 0.13, p = 0.054 for “Do you have a fear of falling while walking?” (Table 3).

**Table 3:** Correlation between sitting time and physical function subitems of KCL number (%) Dependent variable: physical function subitems of KCL Correlation ratio KCL: Kihon Checklist

			Sitting time (η)	Sitting time (η^2^)	p-value
KCL 6. Do you normally climb stairs without using a handrail or wall for support?	Yes	61 (77%)	0.44	0.19	0.045
	No	18 (23%)			
KCL 7. Do you normally stand up from a chair without any aids?	Yes	76 (96%)	0.15	0.02	0.599
	No	3 (4%)			
KCL 8. Do you normally walk continuously for 15 minutes?	Yes	77 (97%)	0.17	0.03	0.472
	No	2 (3%)			
KCL 9. Have you experienced a fall in the past year?	Yes	8 (10%)	0.18	0.03	0.013
	No	71 (90%)			
KCL 10. Do you have a fear of falling while walking?	Yes	17 (22%)	0.13	0.02	0.054
	No	62 (78%)			

## Discussion

The current study determined the association between self-reported sitting time and KCL physical function domain in community-dwelling older adults. Sitting time was associated with the KCL physical function domain score even after adjusting for covariates. Furthermore, the correlation analysis between the KCL subitems and sitting time showed that the length of sitting time was moderately correlated with “Do you normally climb stairs without using a handrail or wall for support?” only.

Sitting time, objectively assessed using a digital device, is associated with decreased physical function [21]. A previous study using a similar device showed that older adults spend approximately 78% of their waking time in a sitting position and that an increase in sitting time affected the decline in walking ability [22]. The development of technology has led to an increase in the use of digital devices to assess PA and sitting time, and it has become accepted that decreased PA and increased sitting time are harmful to health. However, one must bear in mind that such devices are expensive and difficult to manage; thus, they are not a feasible option for everyone. We did not use any digital devices to measure PA and sitting time in our study. Instead, we used self-reported sitting time and assessed physical function using tests and the KCL. The results revealed an association between sitting time and physical function, consistent with previous studies using digital devices. Our findings are significant because physical tests are inexpensive and easy to implement. Similar to ours, a few studies have investigated the association between self-reported sitting time and various negative clinical outcomes. For example, a longer sitting time increases the risk of prefrailty in older adults aged >60 years [10]. Furthermore, longer self-reported sitting time is associated with a higher risk of mortality in older adults [23]. However, these participants were community-dwelling older adults who were surveyed at large. On the other hand, our analyzed cohort comprised health-conscious individuals who exercised regularly and voluntarily participated in physical fitness tests. The finding of an association between sitting time and physical function even in this population reinforces the importance of reduced sitting time for health promotion and preventive care. Recent studies have incorporated digital devices as interventions instead of using them for evaluations [24]. In the future, based on the results of basic observational studies such as ours, self-reporting for the assessment of sitting time and the use of digital devices as intervention methods to reduce sitting time may contribute to health promotion and preventive care among older adults.

Skeletal muscle mass in the extremities of older adults decreases with longer sitting time [25]. Additionally, the risk of sarcopenia increases by 33% for each hour of increased sitting time [26]. Stair climbing is an essential ADL and necessary for independent living. Various physical functions, including walking speed, OLS, and lower limb muscle strength, were reported as related to stair-climbing activity [27,28]. Moreover, stair climbing is an index used to evaluate sarcopenia [29]. Our study results and those of the aforementioned studies suggest that a long sitting time may cause a decline in physical function and make stair-climbing movements more difficult, although our findings are limited because of the cross-sectional design of our study. Considering that difficulty with stair-climbing activities leads to a narrowing of the range of activities and loss of independent ADL [30], our results suggest the need for interventions that reduce sitting time.

The strength of our study is that it is the first to determine the association between self-reported sitting time and the physical function domain of the KCL in community-dwelling older adults. Since our evaluation was easy to perform and did not require specialized equipment, it will contribute to the health promotion and preventive care of older adults based on sitting time. Importantly, our detailed analysis using the KCL physical function subitems revealed an association between sitting time and physical performance in terms of ADL. We believe that this is an important insight into the possibility that the assessment of sitting time and, in turn, intervention in older adults should focus on the specific ADL of stair climbing.

The study also has several limitations that must be considered. First, the analyzed population comprised health-conscious individuals who were recruited from only one region; thus, sampling bias may exist. Second, we were unable to examine the extent of activities other than sitting. Thus, we cannot rule out the possibility that a participant engaged in high-intensity activities even if their sitting time was long or that they engaged in a low amount of other activities even if their sitting time was short. Thus, future studies should consider the amount of activity during waking hours. Third, the number of covariates was limited, and variables potentially associated with physical function and sitting time, such as psychological aspects and nutritional status, should be analyzed. Finally, because this was a cross-sectional study, causal relationships could not be determined. However, this study is significant because it is the first to clarify the association between sitting time and the KCL physical function domain, showing in detail that older adults with longer sitting time tend to have difficulty ascending and descending stairs, thereby contributing to health promotion and preventive care based on PA.

## Conclusions

We demonstrated an association between self-reported sitting time and the KCL physical function domain. Notably, older adults with longer sitting time tended to experience difficulty ascending and descending stairs. The findings underscore the utility of assessing sitting time for maintaining physical function in community-dwelling older adults. We also showed that interventions to reduce sitting time can help maintain and improve physical function. We believe that our study contributes to health promotion and preventive care for older adults based on PA, particularly sitting time.

## Appendices

**Table 4:** Subitem details of KCL This table describes the content of the questions in the subitems of the KCL [15]. KCL: Kihon Checklist, IADL: instrumental activities of daily living

KCL	
IADL	1. Do you go out by bus or train by yourself?
	2. Do you go shopping to buy daily necessities by yourself?
	3. Do you manage your own deposits and savings at the bank?
	4. Do you sometimes visit your friends?
	5. Do you turn to your family or friends for advice?
Physical function	6. Do you normally climb stairs without using a handrail or wall for support?
	7. Do you normally stand up from a chair without any aids?
	8. Do you normally walk continuously for 15 minutes?
	9. Have you experienced a fall in the past year?
	10. Do you have a fear of falling while walking?
Nutritional status	11. Have you lost 2 kg or more in the past 6 months?
	12. Height: cm, weight: kg, BMI: kg/m2 If BMI is less than 18.5, this item is scored.
Oral function	13. Do you have any difficulties eating tough foods compared to 6 months ago?
	14. Have you choked on your tea or soup recently?
	15. Do you often experience having a dry mouth?
Social isolation	16. Do you go out at least once a week?
	17. Do you go out less frequently compared to last year?
Cognitive function	18. Do your family or your friends point out your memory loss? e.g. “You ask the same question”
	19. Do you make a call by looking up phone numbers?
	20. Do you find yourself not knowing today’s date?
Depressed mood	21. In the last 2 weeks have you felt a lack of fulfilment in your daily life?
	22. In the last 2 weeks have you felt a lack of joy when doing the things you used to enjoy?
	23. In the last 2 weeks have you felt difficulty in doing what you could do easily before?
	24. In the last 2 weeks have you felt helpless?
	25. In the last 2 weeks have you felt tired without a reason?
